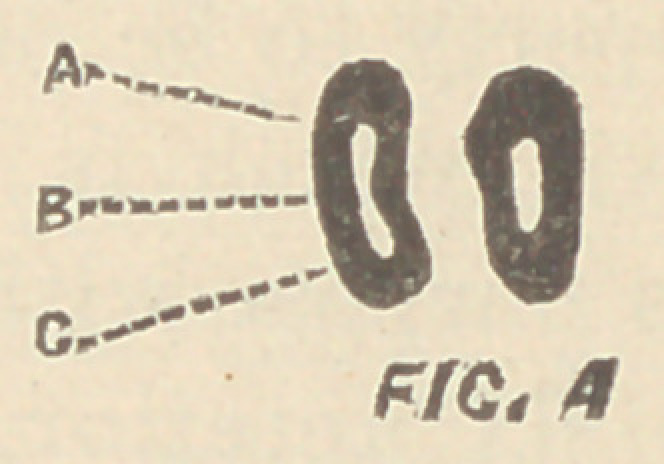# Root Canals Considered in Relation to Filling and Preparation for Artificial Substitutes

**Published:** 1888-11

**Authors:** Louis Ottofy

**Affiliations:** Chicago, Ill.


### ROOT CANALS CONSIDERED IN RELATION TO FILLING AND PREPARATION FOR ARTIFICIAL SUBSTITUTES.

BY LOUIS OTTOFY, CHICAGO, ILL.

READ BEFORE THE NEW JERSEY STATE DENTAL SOCIETY AT ITS EIGHTEENTH ANNUAL SESSION HELD IN ASBURY PARK, THURSDAY EVENING, JULY 19, 1888.

I desire to call your attention to a few points relative to the preparation of root canals for filling or for the attachment of artificial crowns—points of more or less importance, though often under-estimated in dental practice. We are painfully aware that not only the less positive scientific branches, which enter into the study of dentistry, are enshrouded in mist and veiled with a certain amount of indefiniteness and unreliability; but we find that even those branches which are known as the foundation on which the others have been built, are very crude, uncertain, and in many instances entirely false. This is true in some measure of dental anatomy. Many errors in relation to the anatomy of the teeth have crept into early editions on anatomy, or have been culled from works on general anatomy, written or revised by eminent scholars in general medical sciences. The statements have been accepted as facts, and published and republished until these fallacies have become well-grounded opinions of many members of the profession.

It is not so deplorable with those subjects which, instead of being misinterpreted by dental anatomists, have been wholly or partially ignored; because, at least, if the student is not enlightened, he is not misled; but remains in a position to receive correct impressions. Dental anatomists have not given as much attention to the consideration of root canals as their present importance demands. To the dental practitioner of to-day the shape, size and probable length and location of a root canal, and the relative size of the canal to the root, is of the utmost importance—of more importance than at any time in the past. To the dentist of the future much will be clear and concise, which to the busy practitioner of the past and of to-day has been a source of annoyance, and frequently the cause of failure. For the attachment of the wooden peg pivot of the earlier times, whose application was restricted usually to straight-rooted anterior teeth, or for the introduction of fillings into the cavities of pulpless teeth, leaving the roots unfilled (a vent-hole or an abscess affording exit for the putrescent contents of the canal), a limited knowledge as to the root itself, or the channel within it, was sufficient. For the varied operations of root filling and crowns, a better knowledge of the form of root canals is essential, and a thorough understanding of dental anatomy is almost indispensable to the successful practice of dentistry.

The information dental anatomies contain in regard to root canals is generally embodied in the very incomprehensive general expression: "That the pulp chamber and root canals correspond with the general outline of the tooth." In the main this is true, and the perfect knowledge of the anatomy of the teeth seems primarily indispensable. It certainly is essential, though the shape of a root does not always indicate the size, direction or possible bifurcation of its root canals.

That much is to be said in regard to the canals of the upper central incisors; these teeth have perhaps the most favorably-shaped roots of any, and it must be gross carelessness indeed when any manipulation within the pulp-chambers or canals of these teeth do not result favorably. The compression of the roots of these teeth, as so often met with in the lateral incisor, is so rare, that weakening of a frail root while preparing it for the reception of the pivot, or when removing decayed portions therefrom, should never be the result. The form of the root of the lateral incisor is often deceptive; relatively speaking, the root is correspondingly not as wide from the mesial to the distal surface as the root of the central incisor. The crown of the lateral incisor is relatively wider than the crown of the central incisor, and hence there is more liability to weaken the root while enlarging the canal; frequently, also, the root of this tooth is grooved on its flattened side to such an extent that much care must be exercised not to remove too much tooth-substance so as to possibly perforate or weaken the root.

The roots of cuspids require perhaps only the same amount of care bestowed on the central incisors. Their tendency to be flattened laterally should be remembered during the preparation of their canals for the reception of the pivots; neither must one forget the tendency of their roots to bifurcate, as evinced by grooves on their mesial and distal surfaces, and by the frequent division of their canals, which, however, generally unite before reaching the apex of the root.

The form of the root of the first upper bicuspid varies perhaps more frequently than that of any other tooth, about thirty per cent. being bifurcated, and of the remainder but few have a single canal the entire length of the root; most of them have either two partly or entirely distinct canals joining near the apex, and having only one common outlet; or a single-rooted bicuspid may be provided with two canals, each of which has a separate foramen.

The second bicuspid seldom varies, is generally single-fanged, having a single channel, and therefore in its relation to root filling and artificial crown attachment offers no impediments.

In regarding the lower teeth, it should be remembered that the roots of the incisors and cuspids are generally flattened. While the roots are seldom bifurcated, the canals often are bifurcated a part of the distance of their length; but they generally unite and have a common foramen. This is true oftener of the cuspids than of the incisors.

The roots of the lower bicuspids, like those of the upper second bicuspids, offer no impediments to the ready attachment of crowns or the filling of their channels.

It is seldom necessary to introduce posts into the roots of molars, and hence their consideration in that relation is dispensed with.

As a rule the drilling, reaming or any other method of enlarging root canals, is pernicious practice and should be almost entirely abandoned. When a proper entrance to the pulp chamber has been secured, before any effort has been made to enter the root canals, the reaming or enlarging of the canal will not be found necessary. Opening into a pulp-chamber and securing an entrance into a root canal should always be done under perfect antiseptic precautions; for this purpose flooding the cavity with one to two hundred solution of bichloride of mercury has been recommended and found very efficacious. The objections to its use in this way are: its property to corrode the broaches used, thus rapidly destroying instruments, which should always be delicate; and its other property—said to be very marked—of coagulating albumen. Recently I have followed some interesting experiments conducted by Dr. G. V. Black regarding the antiseptic property of essential oils, and observing the property to be possessed in an extraordinary degree by the oil of cassia, which is very diffusible, I have been led to employ it for this purpose with very satisfactory results. In filling the roots of the six anterior upper teeth, difficulties are seldom encountered, except from the occasional small size of the canal in the lateral incisor, which can be readily penetrated by a fine broach, if no foreign substance has been forced into it.

The rule, that over-hanging ledges should be cut away, is inflexible when applied to all the teeth of the mouth, and it may be especially emphasized when the first upper bicuspids are in question; in the treatment of these teeth easy access is invarably necessary. The use of two broaches is good practice; introduce into the canal, which has been found, a large broach (as large a one that can be inserted), and while leaving it there, endeavor to find another canal by means of a fine, stiff, oiled broach.

The upper first and second molars are often the source of much annoyance, because their canals, especially the posterior buccal, are sometimes very difficult to find; frequently the anterior buccal canals are also difficult of approach. It is presumed that free and easy access has been secured. In extreme cases, when the canal cannot be found (generally due to imperfect light), it is best to arrange for a second sitting; if in the meanwhile some diffusible oil, eucalyptol, for instance, has been sealed into the cavity, it is astonishing how readily a canal is found, whose location it was impossible to determine at the previous sitting. From observing the locality in which many drill, bur or cut in looking for the entrance to the posterior buccal root canal, I am led to believe that either our knowledge of dental anatomy is meagre or else we are under the impression that the canals originate from the pulp chamber very much like the legs of an inverted tripod, from the bottom of the seat, in an exact triangle, and hence the opening is searched for too near the posterior and buccal portion of the tooth. In making cross-sections of the molar teeth at the point of bifurcation of the canals, a triangle is never found; on the contrary the tendency is toward a straight line between the palatine and anterior buccal roots as shown in Fig. 1 (root canals of upper first molar), is most often met with.

FIG. 1

FIG. 2

FIG. 3

FIG. 4

The canal will generally be found slightly back of a straight line, between these two and a little nearer to the anterior buccal than the palatine. Fig. 2, which shows the usual entrance to the root canals of upper molars, is a typical case to illustrate this point.

In filling the roots of any of the lower anterior six teeth, their tendency to bifurcate should be remembered. The use of two broaches is a good practice; one broach will often not readily pass to the apex, but a finer one sometimes follows the first broach at the opening of the tooth, passing into the bifurcated portion of the root, then entering the main channel, and passes beyond the first broach to the apex of the root. This is also often the case with the upper first bicuspids.

In filling the roots of the lower bicuspids, their extreme length should be remembered; aside from that, the canals present nothing unusual, are readily accessible, and easily filled.

The manner of treating of the lower first molar, and the position and form of the root canals, is perhaps less perfectly understood than that of any other tooth. It is generally described as a two-rooted tooth. Remembering that the pulp-chamber and root canals have the general outline of the tooth, the supposition that there are but two root canals is very natural. However, we do oftener find three root canals than two. The canal of the posterior root is the most accessible, no matter where the entrance to the cavity, or where the location of the cavity itself is; while ready access to the anterior canals even in badly decayed teeth must generally be obtained by the dentist, if by no other labor, at least by the cutting away of some overhanging ledge. It will then be found that whenever the canal is bifurcated the broach will pass most readily into the anterior buccal root canal, and often the existence of an anterior lingual canal is entirely overlooked. Using three broaches is a good way to determine the location and number of the canals.

Fig. 3 illustrates a cross section of the roots of a lower molar, which is not fully developed, and Fig. 4 the tooth as we usually find it. Generally a broach cannot enter at *b*, and if it has entered a canal at *a*, the other one, marked *c*, will often remain unfilled. This may not be of much consequence when the canals have one common foramen, or when the broach through one of the canals passes to that foramen; but when separate foramina exist —as I have seen them—or when the broach enters the smaller of the two canals, and does not penetrate to the apex, imperfect sealing of it is almost inevitable. In the second molars we do not find this condition, and when access has been secured the filling of the root canals is not fraught with many unfavorable possibilities.

The roots of the third molars above and below are less often treated and filled; but when this is done, the necessary manipulation approximates nearest to that required for the second molars.

The posts of pivot teeth are entirely too large, especially those of the Logan crown; on paper these posts look very well indeed, but if the root is to be cut away sufficiently to accommodate them, there will be either nothing left of it, or at least it will be weakened to a dangerous extent.

In attaching porcelain crowns to the roots of any of the six anterior teeth, whether with or without a band, no unusual difficulties are encountered; but when the first upper bicuspids are to be pivoted, the Dutch biscuit-shaped constriction of the root should be remembered, and also the fact borne in mind that a post can be permitted to extend only for a short distance into the root. In crowning the lower teeth, when using posts, the flattened shape of the six anterior teeth must be remembered.

For the molars above or below all-gold caps are better suited, and the use of posts can generally be dispensed with.

## Figures and Tables

**FIG. 1 f1:**
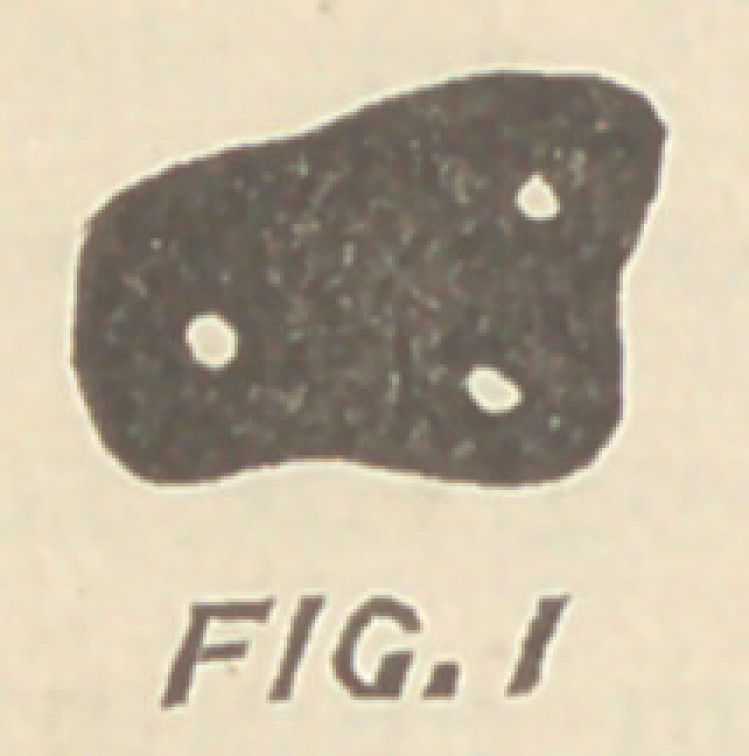


**FIG. 2 f2:**
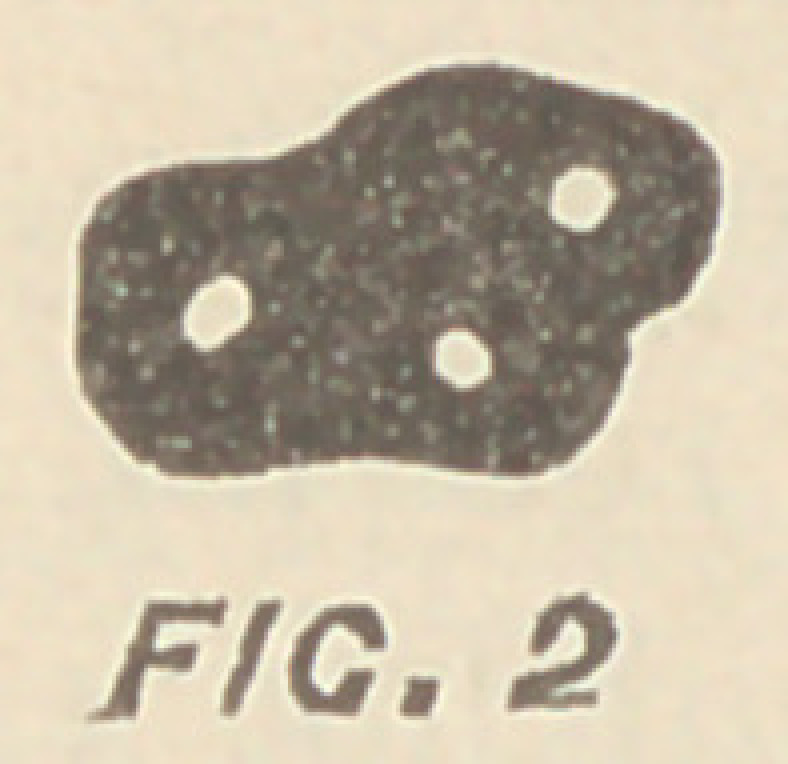


**FIG. 3 f3:**
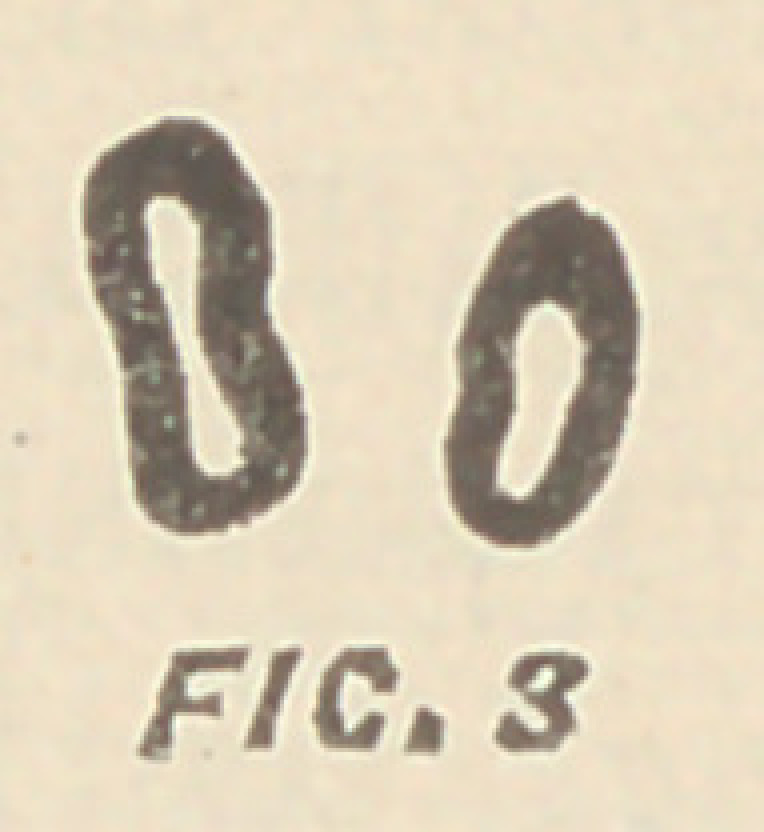


**FIG. 4 f4:**